# Prolactin and 17β-Estradiol Are Epigenetic Regulators That Modify the Effector Response of Bovine Macrophages During *Staphylococcus aureus* Challenge

**DOI:** 10.3390/microorganisms14030576

**Published:** 2026-03-03

**Authors:** Marco Antonio Barajas-Mendiola, Josmarth Remigio-Hernández, Marisol Pérez-Galicia, Joel Edmundo López-Meza, Alejandra Ochoa-Zarzosa

**Affiliations:** 1 Departamento de Biología, División de Ciencias Naturales y Exactas, Universidad de Guanajuato, Noria Alta S/N, Guanajuato 36050, Mexico; ma.barajasmendiola@ugto.mx; 2 Centro Multidisciplinario de Estudios en Biotecnología, Facultad de Medicina Veterinaria y Zootecnia, Universidad Michoacana de San Nicolás de Hidalgo, Km 9.5 Carretera Morelia-Zinapécuaro, Posta Veterinaria, Morelia 58893, Mexico; 1505200g@umich.mx (J.R.-H.); 2132406e@umich.mx (M.P.-G.); elmeza@umich.mx (J.E.L.-M.)

**Keywords:** hormones, macrophages, chemotaxis, phagocytosis, epigenetic mechanisms, *Staphylococcus aureus*, bovine mastitis

## Abstract

*Staphylococcus aureus* (*S. aureus*) is the most prevalent pathogen associated with subclinical mastitis, which significantly impacts dairy farming worldwide. Fluctuations in reproductive hormones, such as bovine prolactin (bPRL) and 17β-estradiol (E2), are known to compromise the innate immune response (IIR) of the mammary gland (MG). In this study, we evaluated the effects of bPRL and E2 on the effector response of primary bovine macrophages, isolated from lactating Holstein cows, challenged with *S. aureus*. We demonstrated that physiological concentrations of bPRL (5 ng/mL) and E2 (50 pg/mL) induced differential changes in the expression of pro-inflammatory (TNF-α, IL-6, and IL-1β) and anti-inflammatory (IL-10) cytokines, chemokines (IL-8), antimicrobial peptides (BNBD10 and S100A7), and miRNAs (miR-451, miR-155, miR-7863, miR-146a, miR-21a, Let-7a-5p, miR-30b, and miR-23a) in *S. aureus*-challenged macrophages. Moreover, these hormones promoted global histone H3 acetylation and the epigenetic H3K9ac mark without affecting H3K9me2 levels. Hormonal treatment also modulated histone deacetylase (HDAC) activity. Furthermore, hormonal treatment altered macrophage chemotaxis and phagocytosis. In conclusion, bPRL and E2 modulate the effector functions of bovine macrophages during *S. aureus* infection. This process could be associated with the regulation of histone H3 modifications, such as H3K9ac, in IIR-related genes.

## 1. Introduction

Subclinical mastitis is a prevalent inflammatory disease of the mammary gland (MG) in dairy cattle, most commonly associated with the Gram-positive bacterium *Staphylococcus aureus*. This pathogen > a major challenge to global dairy production, resulting in substantial economic losses [1,2,3,4,5]. The mammary gland constitutes the primary site for the establishment of *S. aureus* infection [6,7]. Once established, the infection often progresses to a chronic state, weakening the innate immune response and compromising tissue integrity, ultimately leading to functional loss of the MG and, in severe cases, the culling of the animal [8]. Moreover, MG is a complex tissue composed of various cell types (endothelial, epithelial, etc.). It is the target of reproductive hormones such as prolactin (PRL) and 17β-estradiol (E2), which play an essential role during the differentiation, development, and function of this tissue [9]. Furthermore, abrupt changes in these reproductive hormones are associated with IIR imbalance in the MG, establishing a relationship between intramammary infections (IMIs) and the physiological and reproductive status of animals [10,11,12,13].

During peripartum, lactation, and the dry period, cows are more susceptible to IMIs, such as mastitis, and this susceptibility is, in part, associated with reproductive hormones [11,13,14]. As part of the immune response of MG against pathogens, epithelial cells play an important role in triggering a relevant defense against infection because they are in close contact with pathogens [15]. Moreover, our group has explored the role of bPRL and E2 in regulating IIR elements in epithelial cells from MG during *S. aureus* challenge. We demonstrated that bPRL at a physiological concentration (5 ng/mL) induces a pro-inflammatory response and promotes the internalization of *S. aureus* into bovine mammary epithelial cells (bMECs) [16,17,18]. Also, E2 at a physiological concentration (50 pg/mL) induces an anti-inflammatory and antimicrobial response and reduces bacterial internalization [19]. In addition, the hormonal mix causes effects similar to those observed with E2 alone in bMECs challenged with *S. aureus* [20]. However, in bacterial persistence studies, we observed that this response is insufficient to eliminate the infection (personal communication). In this sense, other components of the IIR, such as macrophages, become critical for controlling infection [21].

Macrophages are professional phagocytes that engulf invading microorganisms, such as *S. aureus,* and trigger multiple killing mechanisms to eliminate bacteria efficiently [22]. Among the key effector response mechanisms of macrophages, chemotaxis and phagocytosis are critical for efficient elimination of microorganisms [22]. Despite this, *S. aureus* can survive in macrophages and persist intracellularly, eventually enabling bacterial dissemination [23]. Notably, Alhussien et al. (2015) reported that the number and phagocytic activity of macrophages decreased in animals with subclinical mastitis [24]. The findings demonstrate that chemotaxis and phagocytosis in dairy cattle macrophages are critical processes for animal health. Importantly, several reports indicate that reproductive hormones, such as PRL and E2, can modulate chemotactic and phagocytic activity in macrophages [25,26]. Moreover, a recent report noted that primary cultures of bovine mammary epithelial cells and monocyte-derived macrophages are well-established models for studying epithelial–macrophage interactions during mastitis [27]. However, knowledge of hormone-mediated regulation in bovine macrophages remains scarce, which constitutes one of the main motivations for our study.

On the other hand, epigenetic mechanisms (e.g., DNA and histone chemical modifications, miRNAs) play a significant role in regulating the development, function, and health of the MG [28,29]. In bovines, hypomethylation of the α-casein gene favors its expression, while methylation represses it [30]. Furthermore, an increase in DNA methylation was observed in peripheral blood lymphocytes from cows naturally infected with *S.* aureus and with mastitis [31]. In addition, histone H3K9me3 was upregulated in lymphocytes from cows with subclinical mastitis caused by *S. aureus*, and this was associated with downregulation of inflammatory response genes [32]. Moreover, we previously reported that bPRL and E2 modulate the IIR through H3K9ac and H3K9me2 regulation in bMECs challenged with *S. aureus* [20,33]. miRNAs are another epigenetic mechanism that regulates MG functions [34]. For example, miRNA expression analysis of monocyte-derived macrophages infected with *Streptococcus agalactiae* showed that miRNAs promote an inflammatory phenotype in these cells by regulating the expression of IL-6, IL-1β, and TNFα genes [35]. Notably, miRNAs’ regulatory functions have been widely reported in bovine mammary epithelium, where they act as key regulators of the immune response during bacterial infection [34,36]. Still, their role in bovine macrophage responses is scarce. Although reproductive hormones such as bPRL and E2 are relevant to mammary tissue function, there are no reports on the effects of both hormones on IIR and epigenetic regulation in bovine macrophages during *S. aureus* infection, which was the aim of this work. This research will enable a better understanding of interactions between reproductive hormones and phagocytic cells, opening new therapeutic avenues and improving the well-being and sustainability of dairy herds.

## 2. Results

### 2.1. Multiplicity of Infection (MOI) Determination

**Figure 1:**
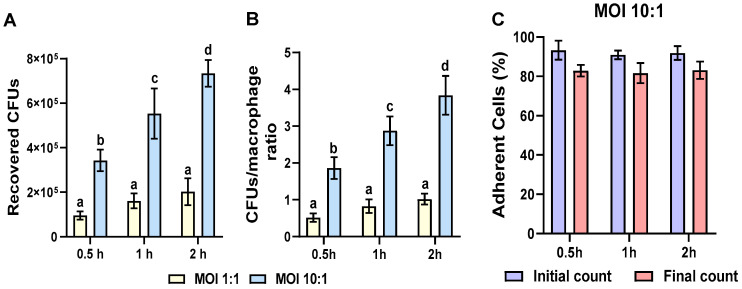
**Determination of multiplicity of infection (MOI) in bovine macrophages challenged with *S. aureus*.** Bovine macrophages (~2.5 × 10^5^ cells) cultured in 24-well plates were challenged with *S. aureus* at different MOIs (1:1 and 10:1, bacteria:macrophage) and at different interaction times (0.5, 1, and 2 h). The gentamicin protection assay was performed, and then CFUs were recovered (**A**). The CFUs/macrophage ratio (**B**) and the viability of infected macrophages (**C**) were determined. Bars represent the mean ± standard error (SE) from three independent experiments (n = 3). Different letters indicate significant differences (two-way ANOVA and Tukey’s post hoc test, *p* < 0.05).

The multiplicity of infection (MOI) is a concept frequently used in infectious disease research, defined as the ratio of infectious agents (e.g., phages, viruses, bacteria, protozoans) to infect a target (e.g., a cell) [37]. Thus, the optimal MOI and the interaction time for evaluating any molecule with immunomodulatory properties should be determined. The results showed that under the experimental conditions reported here, the optimal MOI was 10:1 (bacteria:cell) during the 2 h interaction period, as the recovered CFUs were higher (~4 CFUs/macrophage) and cell viability was ~83% (Figure 1 and Figure S2). Consequently, we selected an MOI of 10:1 and a 2 h interaction time to assess the effects of bPRL and E2 on the effector responses of bovine macrophages during *S. aureus* challenge. Phagocytosis assays were performed for 30 min.

### 2.2. bPRL and E2 Differently Regulate Inflammatory and Antimicrobial Gene Expression in Bovine Macrophages Challenged with S. aureus

**Figure 2:**
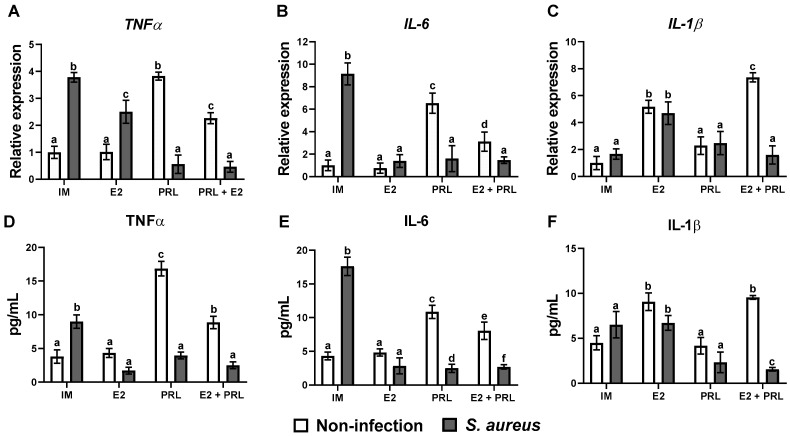
**bPRL and E2 regulate the expression of pro-inflammatory cytokines in bovine macrophages challenged with *S. aureus*.** Bovine macrophages (~2.5 × 10^5^ cells) cultured in a 6-well plate were incubated (24 h) or not with prolactin (bPRL, 5 ng/mL) and/or 17β-estradiol (E2, 50 pg/mL) and then challenged (2 h) or not with S. aureus (MOI 10:1). mRNA expression of TNF-α (**A**), IL-6 (**B**), and IL-1β (**C**) was analyzed by RT-qPCR. The GAPDH gene was used as an internal control of expression for all conditions. Data were normalized with respect to the untreated cell condition (IM: incomplete medium). Bars represent the mean ± standard error (SE) from three independent experiments (n = 3). To analyze cytokine secretion of TNF-α (**D**), IL-6 (**E**), and IL-1β (**F**), supernatants were collected and analyzed by flow cytometry. Bars represent the mean ± standard error (SE) from at least 10,000 events recorded. Different letters indicate significant changes (two-way ANOVA, Tukey’s post hoc test, *p* < 0.05). IM: incomplete medium (untreated cells); bPRL: macrophages treated with bPRL; E2: macrophages treated with 17β-estradiol; bPRL+E2: macrophages treated with the hormonal mix.

To analyze whether the hormones modulate the expression of elements of the IIR in bovine macrophages, we evaluated the expression of pro-inflammatory and anti-inflammatory cytokines, chemokines, and antimicrobial peptide genes by RT-qPCR. Data showed that bPRL, but not E2, significantly induced the expression of *TNF-α* and *IL-6* (3.8- and 6.5-fold, respectively) (Figure 2A,B). A similar effect was observed in cells infected with *S. aureus*; however, this effect was reversed by bPRL (Figure 2A,B). Notably, E2 attenuated the pro-inflammatory effect of bPRL in bovine macrophages because the level of expression of *TNF-α* and *IL-6* was significantly reduced (2.2- and 3.1-fold, respectively) when both hormones were present (Figure 2A,B). Also, in macrophages treated with bPRL or the hormonal mix and then challenged with *S. aureus*, these cytokine expression levels were reduced dramatically (Figure 2A,B). A contrary effect was observed for the *IL-1β* expression; E2, but not bPRL, induced the expression of this cytokine (5.1-fold) (Figure 2C); a similar effect was observed in cells treated with E2 and then challenged with the bacteria (4.6-fold). Also, changes in expression levels were not observed in bovine macrophages treated with or without bPRL or the hormonal mix and then challenged with *S. aureus* (Figure 2C). Notably, the secretion of cytokines coincides with the gene expression of these cytokines (Figure 2D–F). Recently, it was reported that E2 plays an immunoprotective role in murine macrophages against *S. aureus* [38]. In this sense, we evaluated the anti-inflammatory and antimicrobial gene expression in macrophages challenged with *S. aureus*.

**Figure 3:**
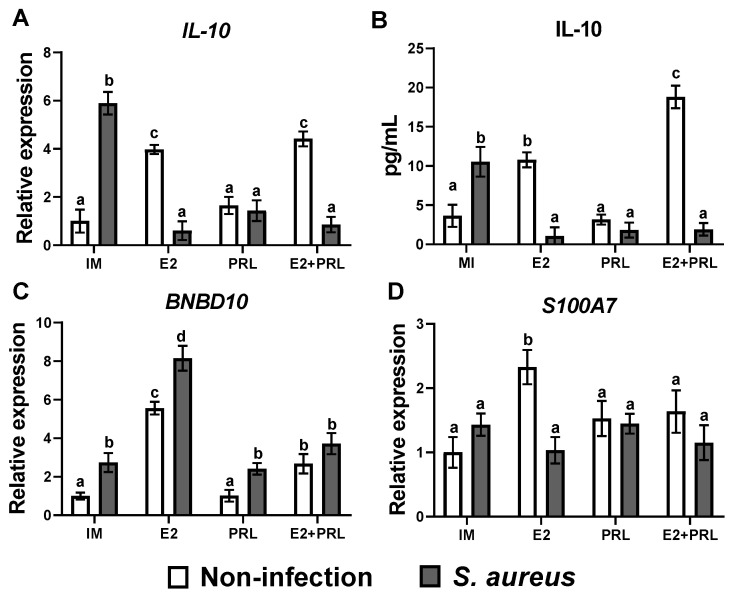
**bPRL and E2 regulate the expression of anti-inflammatory cytokines and antimicrobial peptides in bovine macrophages challenged with *S. aureus*.** Bovine macrophages (~2.5 × 10^5^ cells) cultured in a 6-well plate were incubated (24 h) or not with prolactin (bPRL, 5 ng/mL) and/or 17β-estradiol (E2, 50 pg/mL) and then challenged or not with *S. aureus* (MOI 10:1). mRNA expression of *IL-8* (**A**), *BNBD10* (**C**), and *S100A7* (**D**) was analyzed by RT-qPCR. The GAPDH gene was used as an internal control of expression for all conditions. Data were normalized to the untreated cell condition (IM: incomplete medium). Bars represent the mean ± standard error (SE) from three independent experiments. To analyze IL-8 secretion (**B**), supernatants were collected and analyzed by flow cytometry. Bars represent the mean ± standard error (SE) from two independent experiments. Different letters indicate significant changes (two-way ANOVA, Tukey’s post hoc test, *p* < 0.05). IM: incomplete medium (untreated cells); bPRL: macrophages treated with bPRL; E2: macrophages treated with 17β-estradiol; bPRL + E2: macrophages treated with the hormonal mix.

Moreover, we showed that E2 and *S. aureus*, but not bPRL, significantly induced the expression of *IL-10* (3.9 and 5.8-fold, respectively) (Figure 3A). Notably, when the cells were pre-treated with E2 and then challenged with the bacteria, the expression level was dramatically reduced (0.6-fold) (Figure 3A). Furthermore, when macrophages were treated with the hormonal mix, IL-10 expression was maintained at a similar level to that observed in cells treated only with E2 (4.4-fold); however, in the presence of bacteria, it was dramatically reduced (0.8-fold; Figure 3A). Similar data were observed when IL-10 secretion was evaluated (Figure 3B). Also, E2 and *S. aureus* significantly induced the expression of the antimicrobial peptide *BNBD10* (5.5 and 2.7-fold, respectively) (Figure 3C). Furthermore, cells previously treated with E2 and then challenged with *S. aureus* showed a more accentuated expression of BNBD10 (8.1-fold) (Figure 3C). Notably, the effects observed with E2 were downregulated by bPRL, because macrophages pre-treated with the hormonal mix and then challenged with or without *S. aureus* showed reduced expression of *BND10* (2.6- and 3.7-fold, respectively), which was similar to macrophages challenged with *S. aureus* (Figure 3C). Moreover, E2 significantly induced the *S100A7* expression in macrophages (2.3-fold) (Figure 3D). Additionally, we observed a similar trend for the *BNBD10* gene across all evaluated conditions (Figure 3C). Although *S. aureus* induced a strong activation of macrophages through the TLR2 receptor (Figure S3), the hormonal treatment, in all evaluated conditions, did not affect TLR2 expression in bovine macrophages (Figure S3).

### 2.3. bPRL and E2 Regulate the Production of Nitric Oxide in Bovine Macrophages Challenged with S. aureus

**Figure 4:**
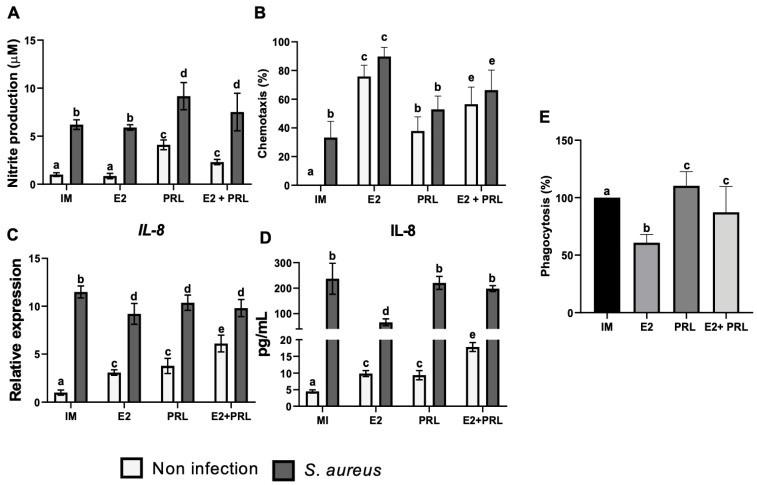
**Effect of bPRL and E2 on elements of innate immune response of bovine macrophages challenged with *S. aureus*.** Bovine macrophages (~2.5 × 10^5^ cells) cultured in a 24-well plate were incubated (24 h) or not with prolactin (bPRL, 5 ng/mL) and/or 17β-estradiol (E2, 50 pg/mL) and then challenged (2 h) or not with *S. aureus* (MOI 10:1). (**A**) The Griess assay was used to analyze nitric oxide production. (**B**) mRNA expression of *IL-8* was analyzed by RT-qPCR. The GAPDH gene was used as an internal control of expression for all conditions. (**C**) IL-8 secretion was analyzed by flow cytometry. Bars represent the mean ± standard error (SE) from at least 10,000 events recorded. (**D**) Chemotaxis assay. Bovine monocytes (2 × 10^5^ cells) were placed in the top of the 24-well Transwell plates, and the conditioned medium obtained from bMECs treated or not with prolactin (bPRL, 5 ng/mL) and/or 17β-estradiol (E2, 50 pg/mL), and challenged (2 h) or not with *S. aureus* (MOI 30:1D), was placed in the bottom. (**E**) Phagocytosis assay. Cells were lysed, and bacteria were grown in LB agar (16 h). Data were normalized relative to the untreated cell condition (IM = incomplete medium). Different letters indicate significant changes (two-way ANOVA, Tukey’s post hoc test, *p* < 0.05). Bars represent the mean ± standard error (SE) from three independent experiments (n = 3). IM: incomplete medium (untreated cells); bPRL: macrophages treated with bPRL; E2: macrophages treated with 17β-estradiol; bPRL + E2: macrophages treated with the hormonal mix.

Additionally, bPRL and *S. aureus*, but not E2, induced nitrite production significantly (4.1 and 6.2 µM, respectively) in bovine macrophages (Figure 4A). Also, E2 attenuated the effect of bPRL in macrophages treated with both hormones, reducing nitrite levels (2.3 µM), but not when macrophages were previously treated with the hormones or the hormonal mix and challenged with *S. aureus* (Figure 4A).

### 2.4. Chemotaxis of Bovine Monocytes Is Regulated by bPRL and E2

Chemotaxis is a key process by which immune cells move to the site of infection to fight against pathogens [22]. We decided to explore whether bPRL and E2 affect chemotaxis in bovine monocytes during an *S. aureus* challenge. We showed that the conditioned medium obtained from the bMECs pre-treated with E2 significantly increased (75%) the chemotaxis of bovine monocytes (Figure 4B); a similar effect was observed in the conditioned medium resulting from the bMECs pre-treated with E2 and challenged with *S. aureus*, where there was a significant increase (89%) of chemotaxis (Figure 4B). Moreover, a significant rise in chemotaxis of bovine monocytes was observed in conditioned medium from bMECs pre-treated with bPRL (37%) and challenged with *S. aureus* (53%) (Figure 4B). Notably, a significant decrease in chemotaxis of bovine monocytes was observed with the conditioned medium resulting from the bMECs pre-treated with the hormonal mix (56%) (Figure 4B). A similar effect was observed in the chemotaxis of bovine monocytes in conditioned medium from bMECs pre-treated with the hormonal mix and then challenged with *S. aureus* (Figure 4B). These findings suggest that E2 induces a potent chemotactic effect on bovine monocytes, whereas bPRL induces a moderate chemotactic effect; however, bPRL attenuates E2’s effect. Furthermore, we showed that bPRL and E2 significantly induced the expression of *IL-8* (3.0 and 3.7-fold, respectively) (Figure 4C). The hormonal combination strongly induced *IL-8* expression (6.1-fold). Moreover, in macrophages (treated or not with the hormones) challenged with *S. aureus*, IL-8 expression was strongly induced (Figure 4C). In agreement with *IL-8* gene expression, we detected a similar result for this cytokine when secretion was evaluated (Figure 4D).

### 2.5. bPRL and E2 Regulate the Phagocytosis of Bovine Macrophages During S. aureus Challenge

Phagocytosis is the key mechanism by which macrophages engulf and destroy pathogens [22]. Importantly, reproductive hormones can modulate phagocytosis by interacting with immune cells, such as macrophages [24,25,26]. Using invasion assays, we evaluated the effect of bPRL and E2 on phagocytosis in bovine macrophages challenged with *S. aureus*. We showed that bPRL considerably increased the phagocytic capacity (110%) in bovine macrophages challenged with *S. aureus* (Figure 4E), while E2 considerably reduced the phagocytic capacity (60%) under the same conditions (Figure 4E). Moreover, when bovine macrophages were pre-treated with the hormonal mix and then challenged with *S. aureus*, a reduction in the phagocytic capacity (13%) was observed (Figure 4E). These findings suggest that bPRL increases phagocytic capacity in bovine macrophages during *S. aureus* challenge, whereas E2 negatively regulates phagocytic capacity and may inhibit the effect of bPRL.

### 2.6. bPRL and E2 Regulate miRNA Expression in Bovine Macrophages During the S. aureus Challenge

**Figure 5:**
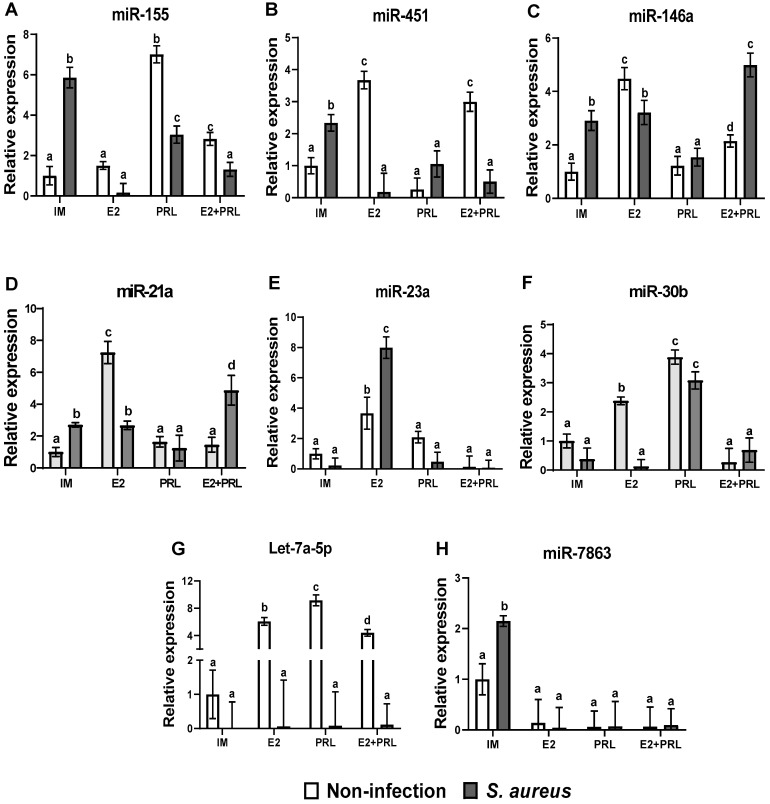
**bPRL and E2 regulate miRNA expression in bovine macrophages challenged with *S. aureus*.** Bovine macrophages (~5 × 10^5^ cells) cultured in a 6-well plate were incubated (12 h) or not with prolactin (bPRL, 5 ng/mL) and/or 17β-estradiol (E2, 50 pg/mL) and then challenged (2 h) or not with *S. aureus* (MOI 10:1). Expression of miR-155 (**A**), miR-451 (**B**), miR-146a (**C**), miR-21a (**D**), miR-23a (**E**), miR-30b (**F**), Let-7a-5p (**G**), and miR-7863 (**H**) was analyzed by RT-qPCR. U6 miRNA was used as an internal control of expression. Data were normalized relative to the untreated cell condition (IM = incomplete medium). Bars represent the mean ± standard error (SE) from three independent experiments (n = 3). Different letters indicate significant changes (two-way ANOVA, Tukey’s post hoc test, *p* < 0.05). IM: incomplete medium (untreated cells); bPRL: macrophages treated with bPRL; E2: macrophages treated with 17β-estradiol; bPRL + E2: macrophages treated with the hormonal mix.

miRNAs are small non-coding RNAs that regulate several cellular functions; their role in the immune response is critical during bacterial infection [39]. In this study, we evaluated miRNAs regulating the IIR by qPCR in bovine macrophages treated with bPRL and E2 and challenged with *S. aureus*. The results showed that miR-155 expression, a master regulator of the inflammatory response, was upregulated by bPRL and *S. aureus* (7.2- and 5.8-fold, respectively) (Figure 5A). However, when bPRL was previously added to the cells and then challenged with the bacteria, there was a significant decrease in the expression level of miR-155 (3-fold) (Figure 5A). Moreover, E2 does not appear to regulate miR-155 expression significantly; however, when cells were pre-treated with both E2 and bPRL, decreased miR-155 expression (2.8- and 1.3-fold, respectively) was observed (Figure 5A). Moreover, a marked decrease in miR-155 expression was observed when cells were infected under the same conditions (Figure 5A). In addition, the results showed that E2 upregulated the expression of miR-451, miR-146a, and miR-21 (3.6, 4.4, and 7.2-fold, respectively) in bovine macrophages (Figure 5B–D). Notably, when the cells were challenged only with *S. aureus*, an upregulation was observed for these miRNAs (2.3-, 2.9-, and 2.7-fold, respectively) (Figure 5B–D). bPRL negatively regulates the effects of E2; when macrophages were pre-treated with the hormonal mix, the level of expression of miR-451, miR-146a, and miR-21a significantly decreased (2.9, 2.1, and 1.8-fold, respectively) (Figure 5B–D). A similar effect was observed for miR-451 in cells pre-treated with the hormonal mix and then challenged with bacteria (Figure 5B). Contrary effects for miR-146a and miR-21a were observed under the same conditions. The hormonal mix favored the upregulation of miR-146a and miR-21a after the challenge with the bacteria (4.9- and 4.8-fold, respectively) (Figure 5C,D). Moreover, we evaluated the miR-23a and miR-30b expression. The results showed that E2, but not bPRL, increased miR-23a expression (3.6-fold), and when cells were challenged with *S. aureus* (post-hormonal treatment), the level of expression was dramatically increased (7.9-fold) (Figure 5E). The expression level of miR-23a was significantly reduced in macrophages treated with the hormonal mix and challenged with or without *S. aureus* (Figure 5E). On the other hand, E2 and bPRL upregulated miR-30b expression (2.3- and 3-fold, respectively); a similar effect was observed in cells previously treated with bPRL and then challenged with *S. aureus* (3-fold) (Figure 5F). Furthermore, the hormonal mix in macrophages, challenged or not with *S. aureus,* dramatically reduced miR-30b expression (Figure 5F). Interestingly, E2, bPRL, and the hormonal mix upregulated Let-7a-5p expression (6.0-, 9.1-, and 4.3-fold, respectively). Still, the expression level of this miRNA was diminished when cells were challenged with *S. aureus* (Figure 5G). Finally, miR-7863 was upregulated in macrophages challenged with *S. aureus* (2.1-fold) (Figure 5H). Interestingly, the hormones abolished the expression of this miRNA in cells challenged with or without *S. aureus* (Figure 5H).

### 2.7. bPRL and E2 Regulate Global Acetylation on the Histone H3 in Bovine Macrophages Challenged with S. aureus

**Figure 6:**
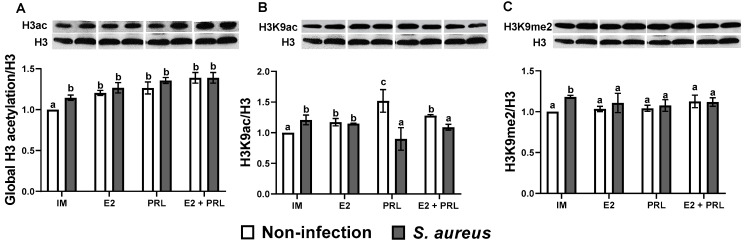
**Regulation of the histone H3 marks by bPRL and E2 in bovine macrophages challenged with *S. aureus* after 12 h of treatment.** Bovine macrophages (~2 × 10^6^ cells) cultured in 6-well plates were incubated (12 h) with prolactin (bPRL, 5 ng/mL) and/or 17β-estradiol (E2, 50 pg/mL), and challenged (2 h) or not with *S. aureus* (MOI 10:1). Densitometric analysis of the immunoblots shows the relative expression of global acetylation of histone H3 (**A**), H3K9ac (**B**), and H3K9me2 (**C**). Data were normalized relative to the untreated cell condition (IM = incomplete medium). Bars represent the mean ± standard error (SE) from three independent experiments (n = 3). Different letters indicate significant changes (two-way ANOVA, Tukey’s post hoc test, *p < 0.05*). IM: incomplete medium (untreated cells); bPRL: macrophages treated with bPRL; E2: macrophages treated with 17β-estradiol; bPRL + E2: macrophages treated with the hormonal mix.

**Figure 7:**
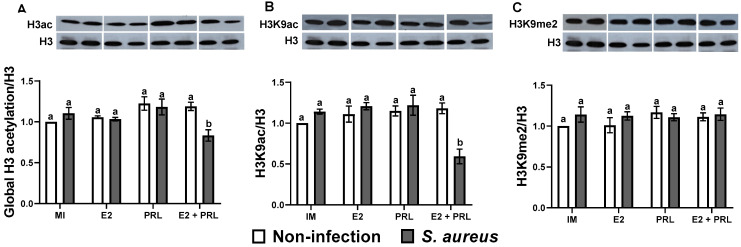
**Regulation of the histone H3 mark by bPRL and E2 in bovine macrophages challenged with *S. aureus* at 24 h of treatment.** Bovine macrophages (~2 × 10^6^ cells) cultured in 6-well plates were incubated (24 h) with prolactin (bPRL, 5 ng/mL) and/or 17β-estradiol (E2, 50 pg/mL), and challenged (2 h) or not with *S. aureus* (MOI 10:1). Densitometric analysis of the immunoblots shows the relative expression of global acetylation of histone H3 (**A**), H3K9ac (**B**), and H3K9me2 (**C**). Data were normalized relative to the untreated cell condition (IM = incomplete medium). Bars represent the mean ± standard error (SE) from three independent experiments (n = 3). Different letters indicate significant changes (two-way ANOVA, Tukey’s post hoc test, *p* < 0.05). IM: incomplete medium (untreated cells); bPRL: macrophages treated with bPRL; E2: macrophages treated with 17β-estradiol; bPRL + E2: macrophages treated with the hormonal mix.

Previously, our workgroup demonstrated important roles of bPRL and E2 in regulating the expression of IIR elements through epigenetic mechanisms using an in vitro model of subclinical bovine mastitis [20,33]. In this sense, macrophages are an important niche for *S. aureus* too [22], and reproductive hormones such as E2 and bPRL can regulate the functions of these cells [14,21]; however, whether epigenetic mechanisms regulate the IIR in bovine macrophages challenged with *S. aureus* remains to be elucidated. The results showed that global acetylation of histone H3 was significantly upregulated in bovine macrophages treated (12 h) with E2, bPRL, and the hormonal mix (1.20, 1.26, and 1.38-fold, respectively) (Figure 6A). A similar result was observed in cells challenged with *S. aureus* (1.14-fold) (Figure 6A). Notably, in cells pre-treated with hormones (E2 or bPRL) separately or with the hormonal mix and then challenged with *S. aureus*, the level of global acetylation of histone H3 significantly increased compared to cells not challenged (1.26, 1.35, and 1.38-fold, respectively) (Figure 6A). However, in macrophages treated for 24 h with hormones, the level of global acetylation of histone H3 was not modified, even when the pre-treated cells were challenged with *S. aureus*, although a significant decrease was observed only in cells previously treated with the hormonal mix and then challenged with the bacteria (0.8-fold) (Figure 7A).

To determine if hormones regulate specific residues on H3, the H3K9ac and H3K9me2 marks were analyzed. The results showed that treatment (12 h) with E2, bPRL, and the hormonal mix significantly upregulated the H3K9ac mark in macrophages (1.17, 1.51, and 1.27-fold, respectively) (Figure 6B). Similarly, in cells challenged with *S. aureus,* an increase in the expression of the H3K9ac mark (1.20-fold) was observed (Figure 6B). Interestingly, in macrophages treated (12 h) with bPRL or with the hormonal mix but not E2, and challenged with *S. aureus*, H3K9ac was significantly decreased at basal levels (Figure 6B). Furthermore, in macrophages treated with hormones for 24 h and challenged with or without bacteria, there were no changes in H3K9ac expression (Figure 7B). Notably, in cells pre-treated (24 h) with the hormonal mix and challenged with *S. aureus,* a significant decrease (0.59-fold) of the H3K9ac mark was observed (Figure 7B). Concerning the H3K9me2 mark, hormonal (separate or mixed) treatment (12 h) did not modify the expression of this epigenetic mark; however, in cells challenged with *S. aureus,* an increase in the H3K9me2 mark was observed (1.18-fold) (Figure 6C). Notably, there were no changes in H3K9me2 at 24 h of evaluation (Figure 7C).

### 2.8. HDAC Activity Is Regulated in Bovine Macrophages Treated with bPRL and E2 and Challenged with S. aureus

**Figure 8:**
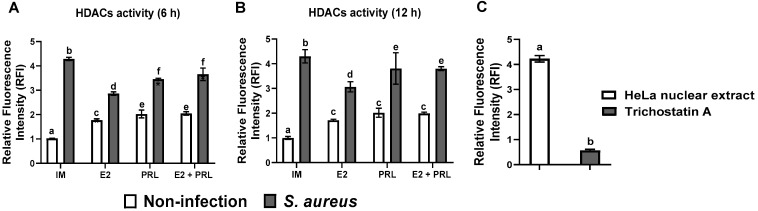
**The activity of HDACs in bovine macrophages treated with prolactin and 17β-estradiol during *S. aureus* challenge.** Bovine macrophages (~2 × 10^6^ cells) cultured in 6-well plates were treated for 6 h (**A**) and 12 h (**B**) with prolactin (bPRL, 5 ng/mL) and/or 17β-estradiol (E2, 50 pg/mL) and challenged (2 h) with *S. aureus* (MOI 10:1). Data were normalized relative to the untreated cell condition (IM = incomplete medium). Bars represent the mean ± standard error (SE) from two independent experiments (n = 2). Different letters indicate significant changes (two-way ANOVA, Tukey’s post hoc test, *p* < 0.05). A HeLa nuclear extract and Trichostatin A were used as positive and negative controls, respectively, of HDAC activity (**C**). IM: incomplete medium (untreated cells); bPRL: macrophages treated with bPRL; E2: macrophages treated with 17β-estradiol; bPRL + E2: macrophages treated with the hormonal mix.

To explore whether bPRL and E2 could modulate H3K9ac by regulating HDACs, the activity of HDAC1 (class I) was evaluated. The HDAC activity was assessed at 6 and 12 h, considering the effects of these hormones on the H3K9ac mark (Figure 6B). Results showed that treatment (6 h) with E2, bPRL, and the hormonal mix in bovine macrophages induced a moderate and significant activity of HDACs (1.17, 2.02, and 2.04-fold, respectively) (Figure 8A). A similar effect was observed in macrophages challenged with *S. aureus* (4.28-fold) (Figure 8A). Notably, in cells previously treated (12 h) with the hormones (bPRL, E2, and hormonal mix) and then challenged with *S. aureus*, the activity of HDACs was increased (2.86, 3.44, and 3.65-fold, respectively) (Figure 8B).

## 3. Discussion

Subclinical mastitis is an inflammatory disease of the mammary gland in dairy cattle primarily caused by the Gram-positive bacteria *Staphylococcus aureus* [2,3,4,5]. Abrupt changes in reproductive hormone concentrations, such as PRL and E2, during peripartum induce an imbalance in the innate immune response of the mammary gland [11,13,14]. Among the IIR components, macrophages are essential for eliminating invading pathogens [40], and their presence in the mammary secretions is a key indicator of tissue health [21,24]. It has been reported that both the number and phagocytic activity of macrophages decrease in cows with mastitis, with a more pronounced effect in subclinical cases [24]. Moreover, the activation of these cells requires timely regulation of gene expression through interactions with transcription factors and epigenetic modifications, which govern macrophage functions [41,42]. In addition, the role of epigenetic mechanisms in regulating macrophage activity during bacterial infection has been described [43]. This work aimed to evaluate changes in the effector response induced by bPRL and E2 in bovine macrophages challenged with *S. aureus,* and to identify the epigenetic mechanisms underlying this response. Firstly, we determined the multiplicity of infection (MOI) in bovine macrophages challenged with *S. aureus*. Our results showed an optimal MOI of 10:1 (bacteria:cell) and an interaction time of 2 h (Figure 1A). Furthermore, the ratio of CFU/macrophage recovered was ~4 bacteria per cell (Figure 1B); these conditions did not affect cell viability (Figure 1C and Figure S2). These results indicate that an MOI of 10:1 and an interaction time of 2 h are appropriate for analyzing the immune response induced by the hormones.

In this sense, we explored the innate immune response induced by E2 and bPRL in bovine macrophages during the *S. aureus* challenge. We showed that bPRL and *S. aureus* induce the expression (Figure 2A,B) and secretion (Figure 2D,E) of the pro-inflammatory cytokines TNF-α and IL-6; this is consistent with reports showing an upregulation of the pro-inflammatory response in alveolar macrophages challenged with *S. aureus* [44], and the upregulation of the pro-inflammatory cytokines induced by PRL [45]. Notably, E2 substantially decreased the expression (Figure 2A,B) and secretion (Figure 2D,E) of TNF-α and IL-6, and the same effect was observed when macrophages were treated with the hormonal mix, even in the presence of *S. aureus* (Figure 2A,B,D,E). This finding suggests that the E2 response could influence the pro-inflammatory response induced by bPRL. Similar results were observed in macrophages from ovariectomized mice challenged with *S. aureus*, where the pro-inflammatory response was inhibited when E2 was added [38].

Although the effects of the hormonal mix in this research may appear additive or nonadditive, we cannot definitively assign these interactions to a single mechanistic level (e.g., receptor competition, pathway interference, or dose/timing effects), given that in mammary glands, these hormones can act alone or in combination, regulating the immune microenvironment [11,13]. However, synergism between PRL and E2 in regulating gene expression has been reported only in human mammary cells [46]. Hence, cross-talk between the PRL and E2 signaling pathways in our experimental model warrants further research. We highlight that our intention here was to understand the role of the hormonal mix in bovine macrophages during *S. aureus* infection. These hormones can be present in cows’ mammary glands in vivo.

Interestingly, we showed that E2 and the hormonal mix, but not bPRL, induce IL-1β expression and production (Figure 1C,F). This agrees with Martínez-Neri et al. [47], who observed in THP-1 macrophages that PRL does not induce the expression or secretion of this cytokine [47]. Importantly, *S. aureus* does not induce the expression and secretion of IL-1β (Figure 1C,F). A similar result was observed in alveolar macrophages infected with *S. aureus* [44]. A possible explanation for this effect is that the Staphylococcal Panton-Valentine Leucocidin (PVL), an important trigger of IL-1β expression [48], was downregulated by the treatment used here. Further research is necessary to address this issue and its implications for inflammasome activation on bacterial clearance [49]. Although IL-1β is produced by macrophages infected with *S. aureus* pre-treated with E2, the presence of the bPRL downregulated this response, suggesting that E2 downregulated the pro-inflammatory response induced by bPRL.

Furthermore, nitric oxide (NO) is produced by iNOS in macrophages and is critical for bacterial elimination [50]. Here, we reported that pro-inflammatory stimuli such as bPRL and *S. aureus* induce NO production in macrophages (Figure 4A). This is in agreement with previous reports demonstrating PRL-induced NO production in peritoneal macrophages [51] and showing that components of the cell wall of *S. aureus* increase NO production in the murine cell line RAW264.7 [52]. Interestingly, the hormonal mix reduced NO production in macrophages but increased it when *S. aureus* was present (Figure 4A). This is consistent with *S. aureus’s* ability to resist nitrosative stress through lactate dehydrogenase (LDH) activity, which is induced by NO and correlates with intracellular bacterial replication [53]. Furthermore, it has been described that NO inhibits the production of pro-inflammatory cytokines such as IL-6, IL-1β, IFN-α, and TNF-α in leukocytes [54], an effect mediated by nitrosylation of the transcription factors JAK/STAT and NF-κB [55]. This fact probably explains how *S. aureus* exploits macrophage immune machinery to downregulate the pro-inflammatory response, and how hormones could contribute to this effect. However, further research is necessary to address this issue. In addition, we showed that both hormones and *S. aureus* upregulated IL-8 expression and secretion (Figure 4B,C), consistent with IL-8 being an important chemokine involved in the migration of leukocytes during bacterial infection [56].

Additionally, *S. aureus* induces the expression and secretion of IL-10 (Figure 3A,B). Similar results were observed in human and mouse monocyte-derived macrophages infected with *S. aureus* [57]. Interestingly, in the same study, the upregulation of IL-10 was associated with bacterial LDH activity [57], which is linked to nitrosative stress resistance in *S. aureus* [53] and correlates with the increase in NO production in our work (Figure 4A). Further investigation is necessary to elucidate this issue. However, E2 and the hormonal mix reduced IL-10 expression in macrophages challenged with *S. aureus* (Figure 4A), in contrast to the study by [57]. possible explanation is that E2 interferes with bacterial LDH activity, thereby reducing IL-10 expression; however, further research is needed to confirm this. Importantly, all effects of the hormones on IIR expression regulation are independent of TLR2 signaling (Figure S3); these effects are likely closely related to JAK/STAT and MAPK signaling pathways [58,59]. Further research is necessary to corroborate this issue.

Antimicrobial peptides, such as cathelicidins and β-defensins, are part of the antimicrobial response of leukocytes, and increased β-defensin gene expression in infected udders confirms their crucial role in the defense of the cow’s mammary gland during mastitis [60]. In this work, we showed that E2 induced BNBD10 expression in macrophages, whether or not they were challenged with *S. aureus*; however, bPRL reduced BNBD10 expression (Figure 3C). Similarly, the upregulation of this defensin was observed in bovine monocytes and neutrophils stimulated with vitamin D. In contrast, in the presence of LPS, the expression of BNBD10 was downregulated [61]. The expression of S100A7 in the alveolus is considered a critical component of the innate immune system and plays an important role in the host mammary gland defense system [62]. In this work, S100A7 was only expressed in macrophages stimulated with E2, while *S. aureus* did not induce the expression of this peptide (Figure 3D). This indicates that S100A7 does not play an essential role in defense of the mammary gland during mastitis caused by *S. aureus*. This is in agreement with a report describing upregulated S100A7 expression in the mammary gland infected with *E. coli* [63].

Epigenetic mechanisms are important regulators of macrophage activity during bacterial infection [42]. miRNAs are small non-coding RNAs (18–24 nt) involved in almost all known cellular processes, including the innate immune system through the modulation of gene expression [64,65]. In this work, we showed that miR-155, miR-451, miR-146a, and miR-21a (Figure 5A–D) are upregulated in macrophages challenged with *S. aureus*; this is consistent with the fact that these miRNAs are involved in the upregulation of the pro-inflammatory genes such as IL-6, TNF-α, and IL-1β, interacting with elements downstream of TLR signaling; moreover, overexpression of miR-155, miR-451, miR-146a, and miR-21a has been observed in intracellular bacterial infections [39,65]. Interestingly, in our study, the overexpression of miR-155, miR-451, miR-146a, and miR-21a coincided with the upregulation of the expression (Figure 2A,B) and secretion (Figure 2D,E) of IL-6 and TNFα, suggesting that these miRNAs could be involved in the regulation of the expression of pro-inflammatory cytokines in bovine macrophages during the *S. aureus* challenge. However, miRNA gain- or loss-of-function assays are necessary to confirm this issue. Notably, the hormones induce the expression of miRNAs in macrophages differentially; while bPRL induces the expression of miR-155 (Figure 5A), E2 induces the expression of miR-451, 146a, and miR-21a (Figure 5B–D). However, the hormonal mix downregulates the expression of these miRNAs, consistent with E2’s anti-inflammatory effects [58]. Interestingly, we report that in macrophages stimulated with the hormonal mix and challenged with *S. aureus*, miR-155 was downregulated (Figure 5A), while miR-146a was upregulated. A similar result was observed by Schulte et al. (2013) in murine macrophages treated with LPS, where the co-expression of miR-155 and miR-146a led to downregulation of the pro-inflammatory response [66]. This is consistent with our findings, where the pro-inflammatory cytokines IL-6, TNFα, and IL-1β were downregulated under these conditions (Figure 2A). In addition, previous reports demonstrated that miR-155 and miR-146a play differential roles in modulating NO production during bacterial infection. Qin et al. (2016) reported that miR-155 downregulates NO production in macrophages infected with *Mycobacterium* [67], while Li et al. (2016) reported that miR-146 upregulates NO production in murine macrophages with BCG [68]. The upregulation of NO production in this work (Figure 3A) coincides with the downregulation of miR-155 and upregulation of miR-155 and miR-146a, respectively (Figure 5A,C). Our results suggest a possible role for these miRNAs in regulating nitric oxide production, likely by modulating inducible nitric oxide synthase expression. Further research is needed to answer this question.

During intramammary infections, circulating hormones such as E2 and bPRL partly activate the mammary epithelium, inducing the secretion of chemotactic factors (e.g., IL-8) that recruit neutrophils and monocytes [11,13,14]. Here, our chemotaxis results showed that supernatants obtained from bMECs pre-treated with a hormonal mix and challenged with or without *S. aureus*, which contain chemotactic elements (e.g., IL-8) [18,19,20], promote the migration of bovine monocytes (Figure 4B).

In addition to their direct effects on monocyte mobilization, hormones can activate mechanisms in macrophages that contribute to chemotaxis. In line with this, we reported that miR-451 is downregulated in macrophages pre-treated with the hormonal mix and challenged with *S. aureus* (Figure 5B), which coincides with upregulation and secretion of IL-8 (Figure 4B,C). This is consistent with Murata et al. (2014), who found that overexpression of miR-451 inhibits neutrophil chemotaxis in vitro and in an in vivo mouse model of rheumatoid arthritis, demonstrating an important role for miR-451 in the pro-inflammatory response [69]. However, further research is needed to correlate miR-451 expression (Figure 5B) with enhanced IL-8 production (Figure 4B,C). This information could be relevant for the expression of chemokines involved in chemotaxis, considering that subclinical mastitis is a chronic inflammatory disease characterized by an increase in somatic cell count, particularly neutrophils [24].

Overexpression of miR-21a has been shown to inhibit the pro-inflammatory response [70], contradicting our results. The downregulation of pro-inflammatory cytokines (Figure 2) does not coincide with the expression of miR-21a (Figure 5D). However, in monocytes infected with *Mycobacterium leprae*, miR-21a inhibits the expression of vitamin D-dependent antimicrobial peptides, suggesting that these bacteria evade the antimicrobial response [71]. This agrees with our findings: the downregulation of miR-21a (Figure 5D) coincides with reduced expression of the antimicrobial peptides BNBD10 and S100A7 (Figure 4C,D). Moreover, bPRL inhibits the effect of E2 and contributes to this effect (Figure 4C,D). This suggests that miR-21a may contribute to the downregulation of E2-dependent antimicrobial peptide expression.

Additionally, we showed that E2 preferentially induces miR-23a expression, whereas bPRL induces miR-30b expression in bovine macrophages, even during the *S. aureus* challenge (Figure 5E,F). We did not find reports of these miRNAs involved in the inflammatory response. However, it has been reported that miR-23a and miR-30b are involved in phagolysosomal maturation during *Mycobacterium tuberculosis* and *Burkholderia pseudomalle* infections, respectively [72,73]. These reports show that downregulation of these miRNAs inhibits phagolysosome maturation in macrophages, a process linked to bacterial infection [72,73]. Although the hormones separately upregulated the expression of miR-23a and miR-30b, even in the presence of *S. aureus*, interestingly, the hormonal mix downregulated the expression of these miRNAs, similarly to macrophages infected with *S. aureus* (Figure 5E,F). This suggests that the hormonal mix could contribute to the inhibition of phagolysosome maturation during bacterial infection in bovine macrophages. Further research is necessary to address this issue. Moreover, Let-7a-5p is involved in downregulating pro-inflammatory cytokines such as IL-6, TNF-α, and IL-1β via TLR signaling [74]. Although the hormones promote the upregulation of Let-7a-5p, its expression does not coincide with the induction of the pro-inflammatory cytokines reported here (Figure 2A–C), possibly because the bPRL and E2 effects are not mediated through TLR signaling (Figure S3). Notably, *S. aureus*, even in the presence of the hormones, downregulated the expression of Let-7a-5p. This agrees with the report by [75]. who demonstrated that Let-7a was downregulated in THP-1 macrophages infected with a small colony variant (SCV) of *Staphylococcus epidermidis,* balancing the pro-inflammatory response with the anti-inflammatory response [75]. However, SCV of *S. aureus* was not confirmed in our study; therefore, further research is needed to corroborate this finding. Interestingly, miR-7863 expression was upregulated only by *S. aureus* in bovine macrophages (Figure 5H), consistent with the report by [75], who proposed this miRNA as a biomarker of both clinical and subclinical mastitis [76]. Moreover, the hormones override the effects of the bacteria (Figure 5H), so it would be interesting to investigate the target of this miRNA and the molecular mechanisms underlying the immune response during bacterial infection. These findings suggest that E2 and bPRL differentially regulate miRNA expression in bovine macrophages.

Likewise, we attempted to correlate the effects of hormones on IIR components with modifications in histone H3 in bovine macrophages during the *S. aureus* challenge. We performed epigenetic analysis at different time points (6, 12, and 24 h) of hormone stimulation because epigenetic effects on chromatin can be achieved in short periods [77]. We showed that after 12 h of treatment, bPRL and E2 induced global acetylation of histone H3 in bovine macrophages (Figure 6A). Furthermore, this mark was maintained in the presence of *S. aureus* (Figure 6A). Notably, at 24 h of hormonal treatment, the global H3 acetylation decreased by ~30% in macrophages challenged with *S. aureus* (Figure 7A). Similar effects were observed in human macrophages derived from peripheral blood monocytes infected with *M. tuberculosis* [78] and in mouse macrophages infected with *Leishmania* amazonensis [79]. Additionally, we analyzed post-translational modifications of histone H3K9, including acetylation and dimethylation. Our results indicate that at 12 h of hormonal treatment, the H3K9ac mark (associated with gene expression) was induced and maintained in the presence of *S. aureus* (Figure 6B). However, the H3K9ac mark decreased ~15% in macrophages pre-treated with the hormonal mix and challenged with *S. aureus* (Figure 6B). Notably, at 24 h of hormonal treatment, the H3K9ac mark decreased by ~50% (Figure 7B) in the same conditions. A similar effect was reported in bMECs treated with bPRL and E2 and challenged with *S. aureus* [20]. This indicates that the changes observed depend on the time of the interaction between cells and hormones. Importantly, these results coincide with the increase in the activity of HDACs, enzymes involved in removing acetyl groups from histones, in macrophages treated (6 h) with the hormonal mix and challenged with *S. aureus* (Figure 8A). Moreover, this effect was maintained at 12 h of the hormonal treatment in the same conditions (Figure 8B). Regarding the H3K9me2 mark (involved in gene repression), only macrophages challenged with *S. aureus* showed upregulation (Figure 6C), whereas hormonal treatment did not alter H3K9me2 levels across the different time points (Figure 6C and Figure 7C). This could be associated with the moderate pro-inflammatory response induced by the hormones, which attenuated G9 activity. This histone methyltransferase catalyzes the methylation of histone 3 lysine 9 (H3K9a), which has been reported to play an important role in alveolar macrophages infected with *Streptococcus* pneumonia [80]. However, if this is occurring in our study model, it requires further research. These findings suggest that the epigenetic H3K9ac and H3K9e2 marks could play an important role in regulating the IIR elements in bovine macrophages; however, this should be corroborated by evaluating their enrichment in the promoter regions of these genes using chromatin immunoprecipitation.

Significantly, epigenetic mechanisms, in addition to regulating IIR gene expression in macrophages during bacterial infections [42], also play essential roles in these cell functions, such as chemotaxis and phagocytosis [81,82]. Here, we showed that both E2 and bPRL induce chemotaxis in bovine monocytes; however, E2 was the most potent (Figure 3). Importantly, this coincides with the downregulation of miR-451, miR-155, and miR-21a expression (Figure 5A–C), which are known modulators of chemotaxis [69,82]. Although we reported here that expression and production of IL-8 (Figure 3B,C), an important chemoattractant during infection, were upregulated by the hormones in our study model, further research is needed to evaluate whether another important molecule, such as CCL2 (Monocyte chemoattractant protein-1, MCP-1), contributes to chemotaxis. Here, we reported that the H3K9ac mark (involved in gene expression) was induced in the presence of the hormones (Figure 6B). However, the H3K9ac mark decreased ~15% in macrophages pre-treated with the hormonal mix. These cells were challenged with *S. aureus* (Figure 6B), which coincides with the increase in HDAC activity (Figure 8) and the decrease in chemotaxis (Figure 4) under the same conditions, likely induced by bPRL. These findings suggest that the hormones may alter the epigenetic H3K9ac mark, an event that likely regulates chemotaxis during *S. aureus* infection. However, this must be corroborated by assessing enrichment of the H3K9ac mark at the promoters of the chemokine genes CCL2 and IL-8 using a chromatin immunoprecipitation assay.

Finally, we decided to evaluate the effect of the hormones on phagocytosis. Here, we showed that bPRL promotes active phagocytosis in bovine macrophages during *S. aureus* challenge (Figure 4E). This is consistent with the fact that macrophages express the PRL receptor (PRLR) [83], and this hormone is associated with phagocytic capacity [26]. Moreover, the effect of E2 may depend on the tissue type and the inflammatory context [38]. Leal et al. (2021) reported that treatment with E2 in ovariectomized mice significantly reduced phagocytic activity in murine macrophages challenged with *S. aureus* [38]; this is consistent with our results, which showed that E2 reduced phagocytic capacity in bovine macrophages during *S. aureus* challenge (Figure 4E). Importantly, it has been shown that overexpression of miR-155 increases phagocytic capacity [84,85], especially in a pro-inflammatory environment, such as PRL, which induces a pro-inflammatory state [45,46]. In this work, we showed that bPRL induced a pro-inflammatory environment (Figure 2A and Figure 3A), overexpressed miR-155 (Figure 5A), and elevated phagocytic activity (Figure 4E) in bovine macrophages challenged with or without *S. aureus*. Importantly, bPRL could promote indirect active phagocytosis by increasing miR-155, which favors M1 polarization and the expression of phagocytic receptors; however, further research is necessary to address this issue. Moreover, there is strong experimental evidence that E2 can induce miR-146a expression in macrophages, thereby reducing their phagocytic activity [85]. This evidence is consistent with our findings, in which we reported that E2 induced a significant overexpression of miR-146 (Figure 5C) and coincided with decreased phagocytic activity (Figure 4E) in bovine macrophages challenged with or not *S. aureus*. Importantly, E2 could reduce phagocytosis by overexpressing miR-146a, which favors M2 polarization; however, further research is needed to confirm this. Moreover, Mombelli et al. [81]. reported that HDAC inhibitors (HDACi) reduced the phagocytic capacity of murine macrophages during *E. coli* and *S. aureus* infection, through the downregulation of the phagocytic receptor Msr1, suggesting that the effect is due to the hyperacetylation of histone H3 [81]. Future research is necessary to correlate the epigenetic mechanisms induced by bPRL and E2 in bovine macrophages during *S. aureus* challenge with phagocytic capacity, because during intramammary infections, such as subclinical mastitis caused by *S. aureus*, the phagocytic capacity of bovine macrophages is reduced [24].

**Figure 9:**
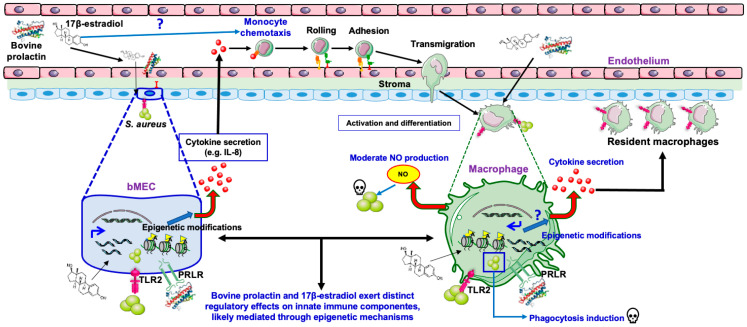
**Conceptual model of hormonal regulation of epithelial–macrophage interactions during bacterial infection.** The figure proposes an integrative model of hormonal regulation of innate immunity, in which epithelial cells and macrophages act as a coordinated system during bacterial infection. Epithelial cells constitute the first physical and immunological barrier and respond to bacterial stimulation by activating signaling pathways and secreting cytokines, including IL-8, which promotes the recruitment of circulating monocytes. Recruited monocytes differentiate into macrophages, where bacterial and hormonal signals induce activation of immune-related genes and production of inflammatory mediators. Hormonal signals modulate epithelial responses and macrophage effector functions through epigenetic mechanisms that regulate gene expression, thereby coordinating innate immune responses. Blue letters indicate the results reported in this work; question mark (?) represents that the indicated effect is not yet elucidated; dead symbol represents bacterial killing. TLR2: Toll-like receptor 2; PRLR: Prolactin receptor; ***S. aureus***: *Staphylococcus aureus*.

Taken together, these findings suggest that reproductive hormones such as E2 and PRL, in addition to participating in the mobilization of monocytes through the chemoattractant molecules (e.g., IL-8) produced by the mammary gland epithelium, also participate in the regulation of the effector response of macrophages (e.g., cytokine production, phagocytosis), which is probably regulated through epigenetic mechanisms (e.g., chemical modification of histones, HDAC activity, miRNA expression) (Figure 9). Furthermore, this contributes to understanding the connection between hormonal regulation, epigenetics, and innate immune defense in the MG during intramammary (IM) disease, such as mastitis.

Moreover, this work describes, for the first time, the effects of reproductive hormones, such as bPRL and E2, on effector responses of bovine macrophages during an *S. aureus* challenge. It remains to be determined whether hormones induce monocyte differentiation, affect resident macrophages, or do both.

This experimental approach allowed us to focus on macrophage intrinsic mechanisms under controlled conditions. The use of primary cells from the natural host, physiological hormone concentrations, and *S. aureus* isolates from clinical mastitis provided biologically relevant insights into innate immune responses. Moreover, primary cultures of bovine mammary epithelial cells and monocyte-derived macrophages represent well-established models for studying epithelial–macrophage interactions during mastitis [27].

## 4. Materials and Methods

### 4.1. Hormones

Purified bovine prolactin (bPRL) (AFP7170E) was provided by A. F. Parlow from the National Hormone and Peptide Program (NHPP)—National Institute of Diabetes and Digestive and Kidney Diseases (NIDDKD) (Torrance, CA, USA) [86]. 17β-estradiol (E2) was purchased from Sigma Aldrich (St Louis, MO, USA). The working solutions were dissolved in sterile water and 1% ethanol, respectively. We used bPRL at 5 ng/mL and E2 at 50 pg/mL in all experiments, as reported [18,19].

### 4.2. Antibodies

For Western blot assays, rabbit polyclonal antibody anti-H3ac (H3K9ac, K14ac, K18ac, K23, and K27ac) (1:1000) (abcam, ab47915, Cambridge, UK), rabbit monoclonal antibody H3K9ac (1:1000) (abcam, ab10812, Cambridge, UK), and mouse monoclonal antibody anti-H3K9me2 (1:1000) (abcam, ab1220, Cambridge, UK) were used as primary antibodies; rabbit polyclonal antibody anti-H3 (1:3000) (abcam, ab1791, Cambridge UK) was used as loading control. Secondary antibodies (1:3000) raised against mouse and rabbit horseradish peroxidase-conjugated antibodies were purchased from Cell Signaling Technology (Danvers, MA, USA).

### 4.3. Staphylococcus Aureus Strain

*Staphylococcus aureus* subsp. aureus (ATCC 27543) strain was used in this work. This strain was isolated from clinical mastitis and can invade mammary epithelial tissue [18]. Bacteria were grown overnight in Luria–Bertani (LB) broth (BIOXON, Becton, Dickinson, México) at 37 °C. Colony-forming units (CFU) were adjusted by measuring the optical density at 600 nm (0.2 OD = 9.2 × 10^7^ CFU/mL).

### 4.4. Primary Culture of Bovine Macrophages

In this study, we used healthy lactating Holstein cows (90–120 lactation days) as donors, collecting 64 blood samples for the trials. Sample collection was authorized by the Faculty of Veterinary Medicine and Zootechnic at the Universidad Michoacana de San Nicolas de Hidalgo (FMVZ-UMSNH) and performed by trained and authorized personnel. All animals used in this study were part of the University’s dairy production herd and had ad libitum access to food and water. Blood mononuclear cells were isolated using the Ficoll-Paque™ Plus system according to the manufacturer’s instructions. Briefly, 4 mL of bovine peripheral blood was mixed with 4 mL of PBS (pH 7.4). The mix was deposited into a 15 mL tube containing 3 mL of Ficoll-Paque™ Plus and centrifuged at 400× *g* at 20 °C for 4 min. The mononuclear cell layer was removed and washed with 4 mL PBS. The cells were centrifuged at 400× *g* at 20 °C for 15 min. The pellet was washed with 4 mL PBS and centrifuged at 100× *g* at 20 °C for 10 min. Mononuclear cells were grown in complete RPMI-1640 medium (Sigma-Aldrich, St Louis, MO, USA) supplemented with 20% fetal bovine serum (FBS) (Equitech Bio, Kerrville, TX, USA), sodium pyruvate 1 mM (Sigma-Aldrich, St Louis, MO, USA), penicillin 100 U/mL (GIBCO, Waltham, MA, USA), streptomycin 100 mg/mL (GIBCO, Waltham, MA, USA), and amphotericin B 1 mg/mL (Sigma-Aldrich, St Louis, MO, USA) (complete RPMI-1640 medium). 2 × 10^6^ mononuclear cells were incubated in 5% CO_2_ atmosphere at 37 °C for 2 h. Then, the floating cells (lymphocytes) were removed, and the adherent cells (monocytes) were washed with PBS. Finally, the monocytes were maintained in complete RPMI-1640 medium in 5% CO_2_ atmosphere at 37 °C for 28 days (renewing the medium every 4–5 days) until differentiation into macrophages (Supplementary Figure S1).

### 4.5. Primary Culture of Bovine Mammary Epithelial Cells

Bovine mammary epithelial cells (bMECs) were isolated from the alveolar tissue of the udder of healthy lactating cows (slaughtered for meat)^16^. Cells from passages 2–8 were used in all experiments. Cells were grown on 90 × 15 mm culture Petri dishes (NEST Biotechnology Co. Wuxi, Jiangsu, China) in Dulbecco’s Modified Eagle’s Medium/Nutrient Mixture F12-Ham (DMEM/F12-Ham) (Sigma Aldrich, St. Louis, MO, USA) supplemented with 10% of fetal bovine serum (FBS) (Equitech Bio, Kerrville, Tx, USA), insulin 10 mg/mL (Sigma Aldrich, St. Louis, MO, USA), hydrocortisone 5 mg/mL (Sigma Aldrich, St. Louis, MO, USA), penicillin 100 U/mL (GIBCO, Waltham, MA, USA), streptomycin 100 mg/mL (GIBCO, Waltham, MA, USA), and amphotericin B 1 mg/mL (Sigma Aldrich, St. Louis, MO, USA) (complete medium). Before use, cells were maintained in 5% CO_2_ atmosphere at 37 °C. All experiments were performed using synchronized cells in DMEM/F12-Ham medium without FBS and antibiotics (incomplete medium) for 24 h.

### 4.6. Multiplicity of Infection Assay

Bovine macrophages were challenged with *S. aureus* at different multiplicities of infection (MOI, 1:1 and 10:1, bacteria:cells) and various interaction times (0.5, 1, and 2 h). Phagocytized CFUs were recovered on agar LB plates after macrophage lysis and maintained at 37 °C for 16 h. CFUs were counted using the Scan 500-Interscience system.

### 4.7. Invasion Assays

Bovine macrophages were cultured in a 6-well plate (NEST Biotechnology Co., Wuxi, Jiangsu, China) previously treated with 200 µL of type 1 rat collagen (Sigma Aldrich, St. Louis, MO, USA). As described above, cells were incubated in complete medium under 5% CO_2_ at 37 °C. Then, cells were treated with bPRL (5 ng/mL) and E2 (50 pg/mL) and incubated in a 5% CO_2_ atmosphere at 37 °C for 24 h. The invasion assay was performed using the gentamicin protection assay as described^16^. Briefly, bovine macrophages treated with bPRL and E2 were challenged with *S. aureus* (OD = 0.2) with an MOI of 10:1 in a 5% CO_2_ atmosphere at 37 °C for 2 h. Next, cells were washed with PBS, and gentamicin at 80 μg/mL was added to eliminate extracellular bacteria for 1 h. Finally, cells were employed to perform the different assays described in the following sections.

### 4.8. RNA Isolation, Gene and miRNA Expression Analysis

To analyze the effect of the hormones on the expression of IIR genes in bovine macrophages challenged or not with *S. aureus*, bovine macrophages (~2.5 × 10^5^ cells) cultured in 6-well plates were incubated with the hormones (24 h) and then challenged or not with *S. aureus* (MOI 10:1). Total RNA (1 µg) was extracted with the Trizol™ Reagent (Invitrogen Waltham, MA, USA) according to the manufacturer’s instructions. The integrity of the RNA was verified by agarose gel electrophoresis, and genomic DNA contamination was removed with DNase I (Invitrogen, Waltham, MA, USA) according to the manufacturer’s instructions. Then, cDNA was synthesized using the GeneScript Reverse™ Transcriptase system (GeneCraft) according to the manufacturer’s instructions. Briefly, 1 µg of total RNA was reverse transcribed to cDNA in a 20 µL reaction containing 5X GeneScript Buffer, 0.5 µg/µL Oligo d(T) (Invitrogen), 10 mM dNTPs mix (Invitrogen, Waltham, MA, USA), 10 mM DTT (Invitrogen, Waltham, MA, USA). The mix was incubated at 80 °C for 3 min and immediately transferred to ice. Finally, 2 U/µL RNAseOUT™ (Invitrogen, Waltham, MA, USA), 25 mM MgCl_2_, and 10 U/µL GeneScript reverse transcriptase were added, and the reaction was incubated at 42 °C for 30 min, followed by 80 °C for 10 min. The RT-qPCR assay was performed using the comparative Ct (ΔΔCt) method on a StepOne Plus Real-Time PCR System (Applied Biosystems, Waltham, MA, USA). The reactions were carried out with qPCR BIO SYBR Green Blue Mix Hi-ROX (Biosystems, Wayne, PA, USA) using specific primers (Elim Biopharm, Hayward, CA, USA). The oligonucleotide sequences and the PCR conditions used in this work were reported in Barajas et al. (2022) [33]. The GAPDH gene was used as an internal amplification control. To analyze the effect of hormones on miRNA expression in bovine macrophages challenged with or without *S. aureus*, the oligonucleotide sequences and PCR conditions used in this work were as reported in Barajas et al. (2022) [33]. U6 miRNA was used as an internal amplification control.

### 4.9. Nitric Oxide Production Assay

The nitric oxide (NO) production was assessed in culture supernatants. It was indirectly measured by the Griess assay, which quantified the nitrite released by bovine macrophages. Briefly, bovine macrophages (~2.5 × 10^5^ cells) were cultured in 24-well plates, incubated with the hormones (24 h), and then challenged or not with *S. aureus* (MOI 10:1). 100 µL of supernatants were placed in 96-well plates, and 50 µL of sulfanilamide solution (Griess reactive A) was added, followed by the addition of 50 µL of N-1-naphthyl-ethylenediamine (Griess reactive B). The reaction was performed at room temperature in the dark for 30 min. Nitrite accumulation was determined by spectrophotometry at 540 nm using a nitrite calibration standard curve.

### 4.10. Analysis of Cytokine Secretion

To analyze the effect of the hormones on cytokine secretion, bovine macrophages (~2.5 × 10^5^ cells) cultured in 24-well plates were treated with the hormones (24 h) and then challenged or not with *S. aureus* (MOI 10:1). The supernatants were collected and used to determine the secretion of cytokines using the BD™ CBA Human Inflammatory Cytokine kit (Fisher Scientific, BD 551811, San Diego, CA, USA), according to the manufacturer’s instructions, and analyzed in a BD Accury™ C6 cytometer (BD Biosciences, Franklin Lakes, NJ, USA).

### 4.11. Histone Extraction and Western Blot Assays

To analyze the effects of the hormones on the epigenetic marks, bovine macrophages (~2 × 10^6^ cells) cultured in 6-well plates were treated with the hormones (12 and 24 h) and then challenged or not with *S. aureus* (MOI 10:1). Then, histone extraction was carried out by acid extraction as reported in [20]. Briefly, cells were washed with PBS and detached with PBS-TE (PBS, 10 mM Tris-HCl, pH 8.0, 1 mM EDTA pH 8.0) on ice. Cells were resuspended in an H-Lysis solution (0.25 M sucrose, 3 mM CaCl_2_, 1 mM Tris, pH 8.0, and 0.5% NP-4) and centrifuged at 3900 rpm at 4 °C for 5 min. Then, the pellet was recovered and washed with H-wash solution (300 mM NaCl, 5 mM MgCl_2_, 5 mM DTT, and 0.5% NP-40) and centrifuged at 3900 rpm at 4 °C for 5 min. Histone extraction was accomplished using H-extraction solution (0.5 M HCl, 10% glycerol, and 0.1 M 2-mercaptoethylamine–HCl). Then, the supernatant was recovered by centrifugation at 13,000 rpm at 4 °C for 10 min, and cold acetone was added (1:5 ratio). Finally, the histones were precipitated for 5 days at −20 °C. The precipitate was recovered by centrifugation at 13,000 rpm at 4 °C for 10 min, dissolved in 25 mL of sterile deionized water, and stored at -80 °C until use. For Western blot analysis, the histones were separated in a 15% SDS-PAGE gel and transferred to a PVDF membrane by semi-dry transfer. The membranes were blocked with 5% non-fat dry milk in cold PBS at 4 °C overnight. Subsequently, the membranes were washed three times with cold TBS and then incubated with the primary antibodies: anti-H3ac (1:1000), anti-H3K9ac (1:1000), anti-H3K9me2 (1:1000), and anti-H3 (1:3000), at 4 °C overnight. Then, primary antibodies were removed and stored at 4 °C. The membranes were washed three times with cold TBS and incubated for 2 h at 4 °C with the horseradish peroxidase-coupled anti-IgG secondary antibody (1:3000). Then, the secondary antibody was removed and stored at −20 °C, and the membranes were washed three times with cold TBS. Finally, 200 mL of ECL substrate was added to the membranes, which were then exposed to an X-ray plate. The plate was manually developed in the dark. Signal intensity was quantified by densitometry using ImageJ 2.14.0 software. Sodium butyrate 3.5 mM was used as a positive control for H3K9ac induction and a negative control for H3K9me2 induction (Supplementary Figure S4). Data were normalized to the H3 signal and presented as relative expression levels compared to untreated cells.

### 4.12. HDAC Activity

Bovine macrophages (~2 × 10^6^ cells) cultured in 6-well plates were treated with the hormones (6 and 12 h) and then challenged or not with *S. aureus* (MOI 10:1). Then, cells were washed and detached with PBS-TE on ice. The samples were immediately prepared for the fluorometric assay according to the manufacturer’s instructions for the histone deacetylase (HDAC Activity Assay Kit) Fluorometric Kit (Abcam, ab15604, Cambridge, UK). The changes in fluorescence intensity were monitored for 1 h in a Varioskan spectrophotometer (Thermo Scientific, Waltham, MA, USA).

### 4.13. Chemotaxis Assay

To analyze the chemotactic capacity of bovine monocytes, we used the Transwell migration assay (Corning^®^ Transwell^®^ 24 plates, Corning, NY, USA) with the supernatant of bMECs treated with hormones and/or not challenged with *S. aureus* as the chemoattractant. First, 2 × 10^5^ bMEC cells were cultured in 24-well plate treated with the hormones (24 h) and then challenged (2 h) or not with *S. aureus* (MOI 30:1). Then, the supernatants were placed in the bottom of the 24-well plate, and 2 × 10^4^ bovine monocytes were placed in the top of the Transwell’s chamber and incubated in RPMI-1640 medium supplemented with 1% FBS in 5% CO_2_ atmosphere at 37 °C for 12 or 24 h to allow migration. Next, the medium in the top of the Boyden’s chamber was discarded, and the membrane was washed with PBS and fixed with 4% formaldehyde for 2 min. Cells that had migrated were stained with diluted crystal violet (1:5) for 10 min. Finally, the number of migrating cells was quantified by bright-field microscopy.

### 4.14. Phagocytosis Assay

Bovine macrophages were cultured in a 24-well plate (NEST Biotechnology Co., Wuxi, China) previously treated with 200 μL of type 1 rat collagen (Sigma Aldrich, St. Louis, MO, USA). As described above, cells were incubated in complete medium under 5% CO_2_ at 37 °C. Then, cells were treated with bPRL (5 ng/mL) and E2 (50 pg/mL) and incubated in a 5% CO_2_ atmosphere at 37 °C for 24 h. The phagocytic activity of macrophages was evaluated using the protocol described in the invasion assays section [16].

### 4.15. Statistical Analysis

The data were obtained from three independent experiments, except for cytokine secretion assays and HDAC activity assays, which were obtained from two independent experiments. Data were analyzed with PRISM 8.0 software using a two-way analysis of variance (ANOVA) and the Tukey’s post hoc test, with a significance level of *p* < 0.05.

## 5. Conclusions

Here, we demonstrate that bPRL and E2 modify the effector response of bovine macrophages during *S. aureus* challenge, and these effects could be mediated by regulating the chemical modifications of histone H3, such as H3K9ac, and/or by altering miRNA expression. To our knowledge, this is the first report to describe the effects of bPRL and E2 as epigenetic regulators that modify the effector response of bovine macrophages during *S. aureus* infection.

## Data Availability

The raw data supporting the conclusions of this article will be made available by the authors, without undue reservation.

## Supplementary Materials

The following supporting information can be downloaded at https://www.mdpi.com/article/10.3390/microorganisms14030576/s1. Supplementary Figure S1: Establishment of the bovine monocyte-derived macrophages primary culture; Supplementary Figure S2: Viability of macrophages challenged with *S. aureus;* Supplementary Figure S3: bPRL and E2 do not induce changes in the TLR2 expression in bovine macrophages challenged with *S. aureus;* Supplementary Figure S4: Positive and negative controls of epigenetic modifications were analyzed by Western blot in bovine macrophages.

## Abbreviations

bPRL	Bovine prolactin
bMECs	Bovine mammary epithelial cells
E2	17β-estradiol
HDACs	Histone deacetylases
H3K9ac	Acetylation of lysine 9 of histone3
H3H9me2	Di-methylation of lysine 9 of histone 3
IIR	Innate immune response
IM	Intramammary
IL	Interleukin
miRNA	microRNA
MG	Mammary gland
MOI	Multiplicity of infection
NO	Nitric oxide
TNF-α	Tumor necrosis factor-alpha
TLR2	Toll-like receptor 2
