# Supplementary material for: Prolactin and 17β-Estradiol Are Epigenetic Regulators That Modify the Effector Response of Bovine Macrophages During Staphylococcus aureus Challenge
Source: Microorganisms. 2026 Mar 3;14(3):576. doi: 10.3390/microorganisms14030576 (PMC13028668; doi:10.3390/microorganisms14030576)
Supplement: Supplementary file 1 [file microorganisms-14-00576-s001.zip › microorganisms-4152192-supplementary.pdf]

Figure S1

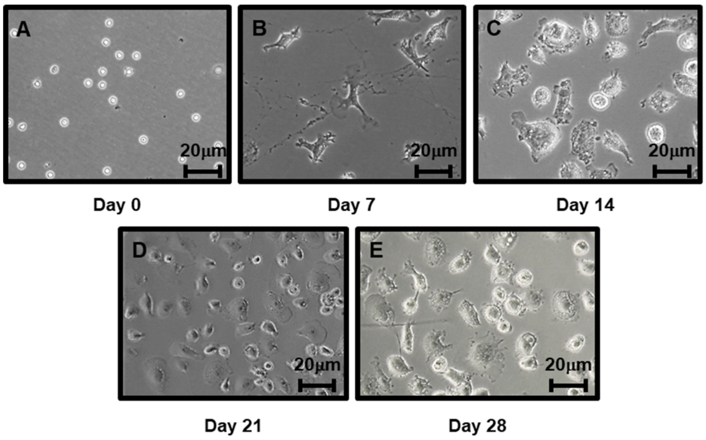

Figure S1. Establishment of the bovine monocyte-derived macrophages primary culture. (A) Representative photomicrographs of bovine monocytes (day 0), **(B-D)** monocyte-macrophage transition, and **E)** differentiated bovine monocyte-derived macrophages (day 28**)** are shown. RPMI-1649 medium plus sodium pyruvate 1 mM and 20% fetal bovine serum was employed for macrophage differentiation. Photomicrographs were taken at 20X magnification. Scale bar= 20 µm.

Figure S2

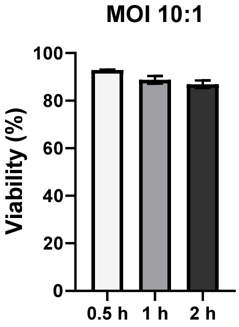

Figure S2. Viability of macrophages challenged with *S. aureus*. Bovine macrophages were cultured in a 24-well plate and then challenged (0.5, 1, and 2 h) with *S. aureus* (MOI 10:1). Cells were recovered by detaching with PBS-TE solution, and viability was analyzed by trypan blue exclusion assay. Bars represent the media ± standard error (SE) from two independent experiments (n=2). Significance was analyzed by One-way ANOVA and post hoc Tukey ($p < 0.05$).

Figure S3

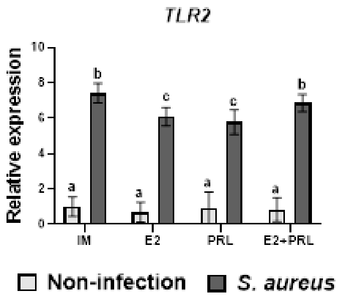

Figure S3. bPRL and E2 do not induce changes in the TLR2 expression in bovine macrophages challenged with *S. aureus*. Bovine macrophages cultured in a 6-well plate were incubated (24 h) or not with prolactin (bPRL, 5 ng/mL) and/or 17β-estradiol (E2, 50 pg/mL), and then challenged (2 h) or not with *S. aureus* (MOI). mRNA expression of *TLR2* was analyzed by RT-qPCR. The GAPDH gene was used as an internal control of expression for all conditions. Data were normalized concerning untreated cells (IM: incomplete medium). Bars represent the media ± standard error (SE) from three independent experiments (n=3). Different letters indicate significant changes (Two-way ANOVA, post hoc Tukey, $p < 0.05$). IM= incomplete medium (untreated cells); bPRL= macrophages treated with bPRL; E2 = macrophages treated with 17 β -estradiol; bPRL + E2 = macrophages treated with the hormonal mix.

Figure S4

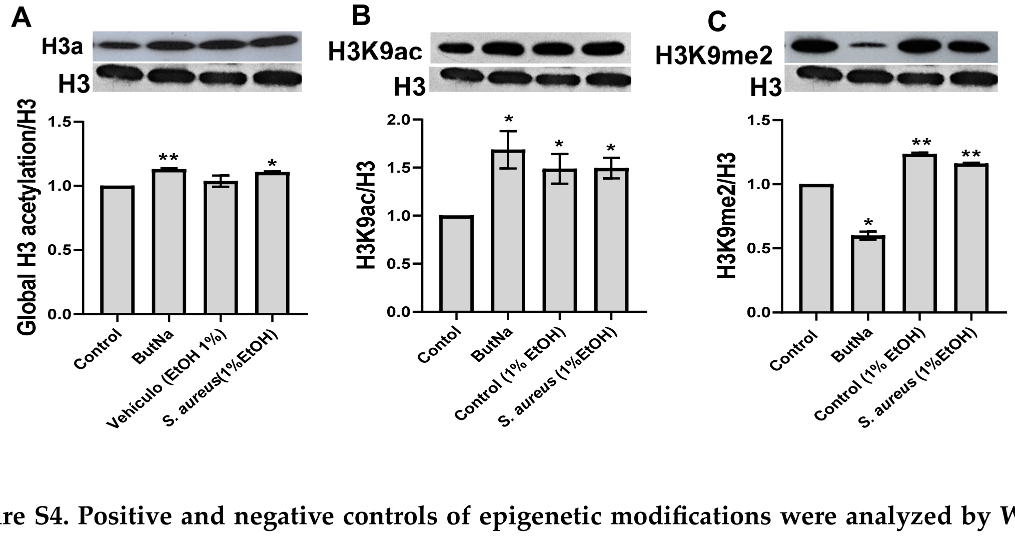

Figure S4. Positive and negative controls of epigenetic modifications were analyzed by Western blot in bovine macrophages. Global acetylation of histone H3 **(A),** H3K9ac **(B),** and H3K9me2 **(C)** from untreated bovine macrophages (**Control**); and treated with 3.5 mM sodium butyrate (ButNa, used as a positive control for acetylation and negative control for methylation). To rule out the effect of the 17 β -estradiol vehicle, bovine macrophages were treated with 1% ethanol (control, 1% EtOH) and then challenged with *S. aureus* (MOI 10:1) (*S. aureus,* 1% EtOH). Bars represent media ± standard error (SE) from two independent experiments (n=2). Values were normalized with respect to the control. "*"($p < 0.05$) and "**"($p < 0.01$) indicate significant changes concerning control (Student's t-test).
